# Optimizing Nutritional Status of Patients Prior to Major Surgical Intervention

**DOI:** 10.14797/mdcvj.1248

**Published:** 2023-08-01

**Authors:** Raul M. Sanchez Leon, Anjana Rajaraman, Mitzi N. Kubwimana

**Affiliations:** 1 Houston Methodist Hospital, Houston, Texas, US

**Keywords:** ERAS®, Enhanced Recovery After Surgery, surgical nutrition guidelines, malnutrition, malnutrition screening, frailty, sarcopenia, perioperative, PONS, perioperative nutrition screen

## Abstract

In patients undergoing elective cardiovascular and thoracic surgery, malnutrition and the deterioration of nutritional status are associated with negative outcomes. Recognition of the contributory factors and the complications stemming from surgical stress is important for the prevention and management of these patients. We have reviewed the literature available and focused on the nutritional and metabolic aspects affecting surgical patients, with emphasis on the recommendations of enhanced recovery protocols. The implementation of enhanced recovery protocols and nutritional support guidelines focusing on the surgical patient as part of a multidisciplinary approach would improve the nutritional status of surgical patients at risk for negative outcomes.

## Introduction

Postsurgical management and prognosis in elective surgery patients is influenced by preoperative nutritional status that predisposes to negative outcomes.^1,2^ Recent initiatives in evidence-based perioperative nutrition guidelines, including enhanced recovery protocols and nutritional support recommendations, aim to provide surgery-focused goals for patient care.

### Background

The American College of Surgeons National Surgical Quality Improvement Program database developed a preoperative mortality predictor (PMP) for general surgery. PMP identified nine variables as predictors of death, among them “inpatient status, poor functional status, age, cancer, comorbidities, and weight loss.”^3^

Hiram Studley in 1936 established the association between preoperative weight loss and postoperative mortality rate in patients with peptic ulcer disease.^4,5^ The understanding of the impact of nutritional status on surgical outcomes showed slow progress until the 2018 joint consensus statement of the Perioperative Quality Initiative (POQI) workgroup and American Society for Enhanced Recovery (ASER).^6^ Since then, recognition of the effect of age, mobility status, comorbidities, presence of malignancies, and performance status on outcomes has promoted the search for evidence-based preventive and therapeutic interventions.^3^

Fowler et al. compared historical references and showed that progressive aging among surgical patients has increased and is expected to continue in the future.^7^ The impact of aging on mechanical muscle function was evaluated by Hvid et al. in young and older healthy active men, comparing muscular function after short-term disuse followed by retraining. After equal time for recovery, younger men showed restored function while older men had an impaired ability to restore function, confirming a similar early study by Suetta et al.^8,9^

Subsequently, establishment of strategies to prevent and address modifiable factors in surgical patients took the form of initiatives such as the Enhanced Recovery After Surgery (ERAS®) program promoted by the ERAS® Society and the European Society for Clinical Nutrition and Metabolism. The American Society for Parenteral and Enteral Nutrition is currently working on ERAS® nutrition guidelines.

## Predictors of Surgical Outcome

The National Veteran Affairs (VA) Surgical Risk Study with the participation of 44 tertiary care VA medical centers and the analysis of a total of 54,215 major noncardiac surgical cases showed the value of albumin levels as a predictor of surgical outcomes, highlighting easy accessibility and low cost.^10^ Meyer et al. evaluated a cohort of 204,819 surgical patients, of which 55.6% corresponded to oncology surgery and 25.4% to cardiovascular surgery. Hypoalbuminemia correlated with worse outcomes, expressed as higher rates of complications, reoperations, readmissions, extended length of stay, and mortality in a cohort of patients undergoing surgical procedures.^11^ A systematic review by Karas et al. in cardiac surgery patients found a correlation with adverse outcomes without establishing a specific level of albumin as an abnormal cutoff.^12^ An evaluation by de la Cruz et al. in 1,164 patients who underwent primary isolated coronary artery bypass graft (CABG) surgery presenting hypoalbuminemia did not show increased early postoperative morbidity or mortality but predicted poor long-term survival after CABG.^13^

Kudsk et al. evaluated patients undergoing non-emergent esophageal and pancreatic procedures with albumin levels below 3.25 g/dL who could have been delayed for nutritional optimization. They were not delayed, and they showed higher risk than colon surgery patients.^14^

Yu et al. used a cutoff of < 20 mg/dL for prealbumin levels to look for the effect on surgical outcomes in cardiac surgery patients. The study found that patients had an increased risk for perioperative infections and extended need for mechanical ventilation support.^15^

While serologic markers and their complementary role for other tools are useful for recognizing clinical malnutrition, it also is important to acknowledge their limitations, as they may overlook a sizable portion of elective surgery patients who otherwise could benefit from interventions aiming at risk modification.^1^

### Nutrition and Frailty in the Surgical Patient

The surgical stress response is a hypermetabolic and hypercatabolic state characterized by protein catabolism, primarily relying on skeletal muscle mass, resulting in a net loss of skeletal muscle mass. Surgeries requiring cardiopulmonary bypass are among the most stressful, and cardiac surgery has the highest rates of iatrogenic malnutrition globally.^16,17,18^ Due to the protein-wasting nature of the surgical stress response, preexisting cachexia, sarcopenia, or malnutrition is a major risk factor for postoperative morbidity and mortality. Impaired functional status is also a major risk factor for poor postoperative outcomes.^19^ Kassin et al. identified that among the factors affecting 30-day hospital readmission in general surgery patients, “failure to thrive/malnutrition” has a frequency of 10.4%.^20^

## Pathophysiological Response to Surgery

The surgical patient presents acute physiological responses to several exposures, including the effect of prolonged fasting, surgical stress response, perioperative hyperglycemia, and enhanced inflammatory response to surgery.

### Preoperative Fasting Effect

Extended preoperative fasting is still prevalent in the preoperative setting despite the wealth of evidence against this practice. The American Society of Anesthesiologists Task Force on Preoperative Fasting and the Use of Pharmacologic Agents to Reduce the Risk of Pulmonary Aspiration issued updated guidelines in 2017. In patients evaluated and cleared for antecedents of gastroesophageal reflux disease, dysphagia, or other motility and metabolic disorders (ie, diabetes), “clear liquids may be ingested for up to 2 hours before procedures requiring general anesthesia, regional anesthesia, or procedural sedation and analgesia.”^21^

In 2003, a systematic review of the effect of different preoperative fasting regimens on perioperative complications and patient wellbeing showed “no evidence that the volume or pH of the participants’ gastric contents differed significantly depending on whether the groups were permitted a shortened preoperative fluid fast or continued a standard fast.”^22^

ERAS® guidelines for clinical nutrition in surgery^23^—including ERAS® Cardiac,^24^ ERAS® Esophagectomy,^25^ and European Society for Clinical Nutrition and Metabolism—all recommend limiting the duration of preoperative fasting.^6,26^

### Surgical Procedure Stress Response

The physiological response to cell injury that surgical intervention entails manifests as an acute stress response. Initial neuroendocrine response with the release of stress hormones, adrenocorticotrophic hormone, cortisol, vasopressin, and growth hormone predispose increased free water retention and hyperglycemia. The effect on glucose homeostasis expressed by increased glycogenolysis and gluconeogenesis, along with insulin resistance, leads to a hyperglycemic state known to increase postoperative complications. It is important to recognize a biphasic hypermetabolic response expressed as an initial ebb phase and flow phase that follows. The ebb starts within hours of the injury and lasts until 2 to 3 days after, manifesting with “a reduction of cardiac output, oxygen consumption, basal metabolic rate, and glucose tolerance.”^27^ The flow phase follows the ebb phase and can last from days to weeks, and it is characterized by an “increase in cardiac output, respiratory rate, oxygen consumption, hyperglycemia, skeletal muscle catabolism, and a negative nitrogen balance.”^27^ As the patient evolves through these phases into a recovery state or a chronic stress state, significant variability occurs in nutrition requirement, intake absorption, and substrate utilization.^27,28^

### Surgery-induced Hyperglycemia

The anabolic effect of insulin promotes glycogenesis and the uptake of glucose into the muscle and adipose tissue. This effect in the context of surgical injury is offset by increased insulin requirements and insulin resistance, leading to an overall catabolic state and residual persistent hyperglycemia. Persistent hyperglycemia in addition to immobilization leads to a multiplying effect of increased insulin resistance and decreased muscular synthesis, thus enhancing the catabolic effect.

Kotagal et al. evaluated a cohort of 40,836 surgical patients from the Surgical Care and Outcomes Assessment Program for diabetes mellitus status, perioperative hyperglycemia, and adverse events. Patients with diabetes had a higher rate of adverse events compared with non-diabetes mellitus patients. Patients with hyperglycemia had an increased risk for events compared with normoglycemic patients, and patients without diabetes mellitus had a dose-response relationship between blood glucose level and adverse events.^29^ The ERAS® guidelines recognize this issue and recommend evaluation of hemoglobin A1c in preoperative evaluation of surgical candidates.

### Surgical Inflammatory Response

The surgical injury triggers a systemic inflammatory response susceptible for being evaluated and followed up by biomarkers such as interleukin-6 and C-reactive protein (CRP). Watt and colleagues in a systematic review involving 14,362 patients undergoing elective procedures showed that interleukin-6 and CRP responses were associated with the magnitude of the operative injury and the invasiveness of the operative procedure.”^4^ Postoperative levels of CRP have shown a strong correlation with complications in patients with major abdominal surgery.^30^ The dual measurement of albumin and CRP in patients with advanced esophageal cancer may offer prognostic parameters in evaluating patients being considered for palliative surgery.^31^

## Malnutrition Screening

Malnutrition screening is the essential first step for identifying patients at risk for malnutrition and will help target patients who would benefit from preoperative nutrition optimization. The Malnutrition Screening Tool used at Houston Methodist Hospital (HMH) is validated in both inpatient and outpatient settings and simply screens for weight loss and eating poorly due to decreased appetite. It does not require a measured height and weight and therefore is quick and easy to administer.^32,33,34,35^

Many other tools exist and are validated with screening criteria of weight status—such as body mass index (BMI) and/or recent weight loss—and recent nutritional intake.^35^ However, none were developed solely for preoperative use. The 2018 joint consensus statement of ASER and the POQI describes a new screening tool, the perioperative nutrition screen, or PONS.^6^ It is a modified version of a previously validated screening tool, the Malnutrition Universal Screening Tool (MUST), which requires a measured height and weight. The MUST assigns risk for low BMI < 18.5, unintentional weight loss, and decreased oral intake.

The PONS changed the BMI cutoff for risk triggers to BMI < 20 and added criteria for age > 62 and low albumin, which as previously discussed has proven to be a valuable surgical prognosticator of postoperative morbidity and mortality. The PONS keeps the original MUST questions regarding unintentional weight loss and decreased oral intake. In the subsequent validation study, unintentional weight loss and low albumin were associated with significantly increased length of stay (LOS) and 30-day readmission rates, while decreased oral intake was associated with significantly increased LOS and low BMI was not associated with increased LOS.^36^ Apart from the PONS, no validated tool for scoring a patient’s perioperative nutritional risk has existed in modern surgical care.^18^

### Nutrition Assessment by Registered Dietitian

Patients who are identified at malnutrition risk should be referred to a Registered Dietitian to perform a formal nutrition assessment along with nutrition-focused physical exam (NFPE) to assess body composition and, if present, to diagnose and quantify degree of malnutrition.^6,36,37,38,39^ Labs are reviewed, including micronutrient levels that need correction, to help with appetite or other symptoms.

### Assessment of Frailty and Sarcopenia

Having identified the malnutrition risk and completed the nutrition assessment and NFPE to diagnose malnutrition, assessment then pivots to identify the extent of frailty in these patients. Handgrip strength using a hand dynamometer and six-minute walk test are both objective and easy to administer.^40,41^ Measured and calculated gait speed also adds to the assessment.^42^ A hand dynamometer is the gold standard for determining decreased functional status, but a simple handshake during the NFPE, where the clinician instructs patients to squeeze tightly, will suffice if these devices are not available.

**Figure 1:**
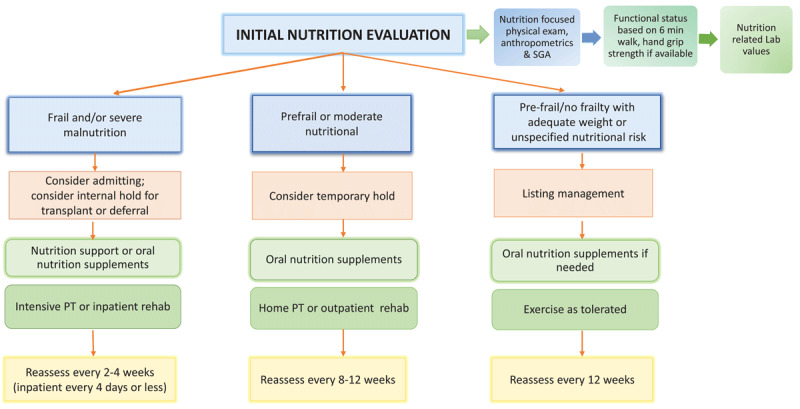
Clinical practice guidelines for frailty followed by the Houston Methodist Hospital outpatient transplant team.

At HMH, the outpatient transplant dietitians use some of these objective measures along with two subjective questions, based on the Fried Frailty Phenotype, to then categorize a patient as frail, pre-frail, or not frail.^43,44^ A treatment plan including nutrition and physical therapy is implemented and, in some cases, surgery is not recommended until progress is made. The algorithm in Figure 1 depicts clinical practice guidelines for frailty that are followed by the HMH outpatient transplant team.

Elderly, obese, and chronically ill frail patients need special consideration of their lean body mass (LBM).^6^ Pairing nutrition assessment and frailty assessment provides insight into a patient’s LBM status, but objective measures of LBM with innovative use of computed tomography and ultrasound for assessment of muscle mass and body composition are emerging in recent literature and are proposed for use in preoperative risk stratification.^6,45^ Weerink et al. evaluated the influence of preoperative sarcopenia using low psoas muscle mass on postoperative complications in surgical oncology patients and showed increased risk of postoperative complications and 30-day mortality.^46^

## Enhanced Recovery After Surgery

The Enhanced Recovery After Surgery (ERAS®) protocol was developed in 1997 as a set of interventions aimed to improve outcomes in patients undergoing elective surgery. The protocol encompasses a series of interventions preadmission, preoperative, intraoperative, and postoperatively. Table 1 highlights ERAS® nutrition-related recommendations for general surgery and subspecialized populations of interest to our readers (cardiac, lung, and esophagectomy).^23,24,25,47,48^

**Table 1:** The Enhanced Recovery After Surgery nutrition-related recommendations for general surgery and subspecialized populations including cardiac, lung, and esophagectomy patients.


Preadmission	Preoperative nutritional screening and assessment plus nutritional support Esophagectomy: in high-risk cases, preference to enteral support by feeding tubes Optimization of chronic disease (identification of hyperglycemia and optimization of diabetes mellitus)

Preoperative	Preoperative carbohydrate loading Cardiac surgery: minimize fasting by continuing clear liquids up to 2 to 4 hours before anesthesia

Intraoperative	Maintenance of fluid balance avoiding both under- and over-hydration Esophagectomy: avoid positive balance resulting in weight gain > 2 kg/d

Postoperative	Early intake of fluids and solids Intake of nutritional supplements Cardiac surgery: insulin infusion in all patients with postoperative hyperglycemia Esophagectomy: enteral feeding with nutritional rate on target by day 3-6


A systematic review of the effect of enhanced recovery programs (ERP) on surgical patients in different surgical specialties showed that ERP decreased the length of stay and the risk for complications within 30 days.^49^

The implementation of an ERAS® cardiac program in the United States was evaluated by Williams et al. and demonstrated improved outcomes expressed as decreased LOS, decreased total intensive care hours, and gastrointestinal complications.^50^

The use of oral nutritional supplements (ONS) providing preoperative carbohydrate load (PCL) is intended to blunt a hyperglycemic surge associated with fasting and surgical stress. The intervention uses ONS such as oral carbohydrate-loading mixtures, immunomodulatory formulas, and isocaloric and/or high-protein supplements. Additionally, enteral and parenteral nutrition support is used in selected patients. Smith et al. performed a systematic review evaluating the effects of PCL compared with placebo on preoperative fasting, postoperative recovery, and insulin resistance in patients undergoing elective surgery. The results showed that PCL was associated with shortened length of hospital stay and did not show aspiration pneumonia in any of the study groups.^51^

Gianotti et al. evaluated the effect of PCL in patients with elective abdominal surgery and showed that PCL effectivity maintained glycemia < 180 mg/dL without affecting the risk for postoperative complications.^52^ Feguri et al. studied the use of PCL and IV omega-3 polyunsaturated fatty acids in 57 patients undergoing CABG and evaluated postoperative outcomes. The results showed no effect on LOS in the ICU or total days but did show a significant decrease in postoperative atrial fibrillation.^53^ The guidelines of the Endocrine Society for the management of hyperglycemia in adult hospitalized patients discourage PLC for patients with type-one diabetes, type-two diabetes, or other forms of diabetes when undergoing surgical procedures due to “potential for harm and uncertainty of benefit.”^54^

The pre- or postoperative use of immunomodulatory (IMN) formulas—containing most commonly arginine, omega-3 fatty acids, and antioxidants delivered by ONS—or enteral nutrition formulas aim to reduce risk in surgical patients. The weight of the evidence supporting their use is variable, depending on the surgical population studied. They generally are accepted in patients at high nutritional risk or who present with underlying malignancies and benefit from a decreased incidence of infectious complications.^6,55^ In opposition, their use is not supported by the ERAS®-Esophagectomy protocol due to lack of significant evidence.^25^

Braga et al. evaluated patients with malnutrition as candidates for major elective surgery due to malignancy of the gastrointestinal tract. Patients were divided into preoperative, perioperative, and control groups and were provided an IMN formula, with all showing a significantly shorter LOS.^56^ Tepaske et al. evaluated high-risk patients undergoing elective cardiac surgery to randomly receive preoperative IMN or standard ONS to evaluate effect on host defense and showed significant decrease in IL-6 and higher expression of HLA-DR in the IMN intervention group along with reduction in total infectious complications.^57^

In a study by Senkal et al., 154 patients eligible for elective surgery due to malignancy of the upper gastrointestinal tract were randomized to oral IMN versus isoenergetic control diet preoperatively, and via catheter jejunostomy postoperatively, and demonstrated decreased occurrence of early infectious complications as well as cost reduction related to treating complications.^58^ An umbrella review by Slim et al. evaluating perioperative IMN versus normal diet or isocaloric isonitrogenous feeding in abdominal surgeries (including esophageal) found significantly fewer infectious complications and less postoperative morbidity.^59^

Patients undergoing cardiac surgery have a distinctive hyperinflammatory response to surgical stress. Leong et al. compared a cohort of patients undergoing elective CABG and/or valve surgery and randomly receiving oral “metabolic therapy” (coenzyme Q, magnesium orotate, lipoic acid, omega 3 fatty acids, and selenium) versus placebo, and they showed improved redox status and myocardial damage, and shortened length of postoperative hospital stay.^60^

### Nutritional Interventions

Pharmaconutrients are indicated as a complement or substitute of oral nutrition in patients at risk for malnutrition or malnourishment. In the surgical population, the prevalence of malnutrition is increased due to “undernutrition” associated with extended nil per os orders and increased nutritional requirements from the surgical stress and heightened catabolic state. Oral nutrition should always be considered early and first, if possible, in the postoperative period.^2,55^ In an international and multi-institutional study, Heyland et al. showed that when enteral feeding is started late, 38.8 hours after admission, patients received only 61.2% of calories and 57.6% of protein prescribed, with 74% of patients not meeting at least 80% of their energy target.^61^ Undernutrition is particularly present in cardiac surgery^62,63^ and in patients subject to esophagectomy. Patients undergoing esophagectomy could remain nutritionally compromised in the months/years that follow and may never return to baseline.^64^

### Oral Nutrition Supplementation

Oral nutrition supplements are indicated for patients with a preserved functional and structural intact gastrointestinal tract that cannot meet adequate nutritional goals for their calculated or measured requirements. The formulations vary from standard to caloric dense and standard to high protein formulations. IMN formulas are defined as containing arginine, omega-3 fatty acids, and a complex carbohydrate such as maltodextrin. IMN formulations have been recommended in the preoperative and postoperative period in patients undergoing elective surgery.^23,24,25,47,48^ Berkelmans et al. performed an international multicenter trial with patients undergoing minimally invasive esophagectomy, testing direct start of oral feeding versus standard of care and the effect on primary (functional recovery) and secondary outcomes (anastomotic leak, pneumonia rate, and surgical complications). It showed no compromise of functional recovery without an increased incidence of postoperative complications.^65^

### Enteral Nutrition

Enteral nutrition (EN) is indicated in patients unable to meet nutritional requirements due to functional or structural limitations. In the preoperative period, EN could be a complement in patients with suboptimal oral intake, limited compliance with ONS, and patients at high risk of malnutrition or malnourished as part of the preoperative optimization.^66,67^ In the postoperative period, EN could be started after admission to intensive care^68^ and increased stepwise to achieve the nutritional goal over 3 to 5 days.^26,55^ Berger et al. studied intestinal absorption in hemodynamic unstable cardiac surgery patients using an acetaminophen absorption test and showed decreased (but not suppressed) absorption associated with decreased pyloric motility.^69^ The use of early EN is supported^68^ in critically ill and surgical patients alike: in a systematic review of a hospitalized mixed-population, Marik and Zaloga showed that early EN was associated with a significantly lower incidence of infections and reduced hospital LOS.^70^ In a group of patients with upper gastrointestinal cancer (54 esophageal, 38 gastric, 29 pancreatic), Barlow et al. randomly assigned patients to EN versus control (nil per os and IV fluid) and showed that EN was associated with shortened LOS and improved outcomes.^66,71^

An earlier study by Gabor et al. evaluated the impact of early postoperative EN on patients with esophagectomy or esophagogastrectomy and reconstruction and found that they recovered faster in terms of ICU and hospital stay, with no impact on mortality.^72^ A major drawback of this intervention is limited compliance and inappropriate quantification of delivery of the nutritional prescription.

EN is the first choice of nutritional delivery in all patients able to tolerate it. Li et al. performed a meta-analysis of 10 studies evaluating safety and efficacy of jejunostomy versus nasoenteric tube in patients undergoing esophagectomy. Patients in the jejunostomy group had a lower incidence of postoperative pneumonia, shorter LOS, and lower risk for catheter dislocation, placing jejunostomy as the preferred EN route in these patients.^73^

Critically ill patients are often underfed due to interruptions in EN delivery. Interventions to improve delivery include the use of energy-dense formulations and strategies such as volume-based feeding, which allow for a catch-up time. The use of energy-dense formulation (1.5 kcal/mL versus 1.0 kcal/mL), evaluated by the TARGET Investigator Group, did not affect mortality (primary outcome) but showed the delivery of similar volumes of EN in both groups, with increased calorie delivery in the 1.5 kcal/mL subgroup.^74^ In the surgical critically ill patient, undernutrition is prevalent along with an increased frequency of parenteral nutrition (PN),^62^ raising the question of benefit of PN over EN. Mazaki and Ebisawa studied the benefit of EN over PN in trials that included 2,552 patients after elective gastrointestinal surgery and found a reduction of “any complications, infectious complications, anastomotic leak, intraabdominal abscess and duration of hospital stay.”^75^

### Parenteral Nutrition and Supplemental Parenteral Nutrition

The use of PN is indicated in the perioperative period in surgical patients who are unable to meet nutritional requirements with oral intake or EN. PN is indicated in the well-nourished patient after 7 days of admission if EN is delivering < 50% of the caloric requirements.^26^ In the postoperative period, use of PN is indicated if EN is deemed high risk for complications (ie, high vasopressor requirements) or gastrointestinal intolerance prevents adequate nutritional delivery, and in those patients with severe malnutrition or high nutritional risk.

Supplemental parenteral nutrition (SPN) is indicated to complement EN when it is insufficient to deliver caloric requirements, always keeping EN as tolerated, to preserve gastrointestinal trophism. Heidegger et al. evaluated the effect of SPN in 153 patients targeting at 100% of energy delivery from day 4 to 8 upon admission to the ICU; they found that patients randomized to SPN versus EN had a lower occurrence of nosocomial infections after day 8.^76^ A similar study by Wischmeyer et al. showed no increase in infection risk in patients receiving SPN.^77^

The evolution of intravenous lipid emulsion (ILE) development and the impact on outcomes in patients receiving PN is noteworthy, from the historical use of soy-based emulsions to the latest incorporation of ILEs with a mixture of olive oil-based emulsion and fish oil (FO)-based emulsions. Manzanares et al. performed a systematic review and meta-analysis evaluating the efficacy of FO-based emulsions on clinical outcomes in critically ill patients. Ten randomized controlled studies including 733 patients were evaluated for clinical outcomes and showed that FO-based emulsions may be associated with a reduction in infections.^78^

## Special Populations

### Extracorporeal Membrane Oxygenation

Extracorporeal membrane oxygenation (ECMO) is indicated in critically ill patients with cardiac or respiratory failure, and the use of EN in this population has been a subject of concern for practitioners. Ferrie and colleagues evaluated 86 patients retrospectively who received ECMO at a single center: 31 patients receiving ECMO for heart failure were subject to venoarterial (VA) ECMO, with the rest receiving venovenous ECMO. All received early EN, and it was well tolerated with both modalities.^79^ Umezawa et al. showed feasibility and safety in a case series of seven patients receiving VA ECMO with nutrition support for severe hemodynamic failure using early EN in a step-up manner over 4 days.^80^ Karpasity reviewed published randomized trials and observational studies between July 2000 and July 2020, evaluating nutritional interventions in critically ill patients receiving ECMO therapy for nutritional adequacy and gastrointestinal complications. EN was found feasible and safe albeit with difficulties meeting nutritional goals.^81^

## Summary

In patients undergoing elective surgery, failure to identify, quantify, prevent, and intervene with proper perioperative non-nutritional and nutritional interventions leads to negative outcomes. The evidence supporting the use of enhanced recovery nutrition protocols applies to most patients and is best implemented in a multidisciplinary and integrative approach. Development of higher quality evidence in this area would increase the applicability in specific surgical groups.

## Key Points

- Screen all patients for malnutrition risk before major cardiothoracic interventions. Implement the use of PONS, a new validated perioperative nutrition screen, in all patients undergoing major cardiothoracic interventions.
- Quantify degree of malnutrition and frailty and incorporate a multidisciplinary integrative approach to therapeutic interventions.
- Adapt evidence-based ERAS® (Enhanced Recovery After Surgery) protocols whenever possible for all planned surgeries.

## References

[1] Portuondo JI, Probstfeld L, Massarweh NN, et al. Malnutrition in elective surgery: How traditional markers might be failing surgeons and patients. Surgery. 2020 Dec;168(6): 1144-1151. doi: 10.1016/j.surg.2020.08.012 32919780

[2] Lobo DN, Gianotti L, Adiamah A, et al. Perioperative nutrition: Recommendations from the ESPEN expert group. Clin Nutr. 2020 Nov;39(11): 3211-3227. doi: 10.1016/j.clnu.2020.03.038 32362485

[3] Vaid S, Bell T, Grim R, Ahuja V. Predicting risk of death in general surgery patients on the basis of preoperative variables using American College of Surgeons National Surgical Quality Improvement Program data. Perm J. 2012 Fall;16(4): 10-7. doi: 10.7812/TPP/12-019 PMC3523928 23251111

[4] Studley HO. Percentage of weight loss: a basic indicator of surgical risk in patients with chronic peptic ulcer. 1936. Nutr Hosp. 2001 Jul-Aug;16(4):141-3; discussion 140-1. 11680474

[5] Parekh NR, Steiger E. Percentage of weight loss as a predictor of surgical risk: from the time of Hiram Studley to today. Nutr Clin Pract. 2004 Oct;19(5): 471-6. doi: 10.1177/0115426504019005471 16215141

[6] Wischmeyer PE, Carli F, Evans DC, et al. Perioperative Quality Initiative (POQI) 2 Workgroup. American Society for Enhanced Recovery and Perioperative Quality Initiative Joint Consensus Statement on Nutrition Screening and Therapy Within a Surgical Enhanced Recovery Pathway. Anesth Analg. 2018 Jun;126(6): 1883-1895. doi: 10.1213/ANE.0000000000002743 29369092

[7] Fowler AJ, Abbott TEF, Prowle J, Pearse RM. Age of patients undergoing surgery. Br J Surg. 2019 Jul;106(8): 1012-1018. doi: 10.1002/bjs.11148 31115918

[8] Hvid LG, Suetta C, Nielsen JH, et al Aging impairs the recovery in mechanical muscle function following 4 days of disuse. Exp Gerontol. 2014 Apr; 52:1-8. doi: 10.1016/j.exger.2014.01.012 24447828

[9] Suetta C, Hvid LG, Justesen L, et al. Effects of aging on human skeletal muscle after immobilization and retraining. J Appl Physiol (1985). 2009 Oct;107(4): 1172-80. doi: 10.1152/japplphysiol.00290.2009 19661454

[10] Gibbs J, Cull W, Henderson W, Daley J, Hur K, Khuri SF. Preoperative serum albumin level as a predictor of operative mortality and morbidity: results from the National VA Surgical Risk Study. Arch Surg. 1999 Jan;134(1): 36-42. doi: 10.1001/archsurg.134.1.36 9927128

[11] Meyer CP, Rios-Diaz AJ, Dalela D, et al. The association of hypoalbuminemia with early perioperative outcomes - A comprehensive assessment across 16 major procedures. Am J Surg. 2017 Nov;214(5): 871-883. doi: 10.1016/j.amjsurg.2016.11.023 29106849

[12] Karas PL, Goh SL, Dhital K. Is low serum albumin associated with postoperative complications in patients undergoing cardiac surgery? Interact Cardiovasc Thorac Surg. 2015 Dec;21(6): 777-86. doi: 10.1093/icvts/ivv247 26362629

[13] de la Cruz KI, Bakaeen FG, Wang XL, et al. Hypoalbuminemia and long-term survival after coronary artery bypass: a propensity score analysis. Ann Thorac Surg. 2011 Mar;91(3): 671-5. doi: 10.1016/j.athoracsur.2010.09.004 21352977

[14] Kudsk KA, Tolley EA, DeWitt RC, et al. Preoperative albumin and surgical site identify surgical risk for major postoperative complications. JPEN J Parenter Enteral Nutr. 2003 Jan-Feb;27(1):1-9. doi: 10.1177/014860710302700101 12549591

[15] Yu PJ, Cassiere HA, Dellis SL, Manetta F, Kohn N, Hartman AR. Impact of Preoperative Prealbumin on Outcomes After Cardiac Surgery. JPEN J Parenter Enteral Nutr. 2015 Sep;39(7): 870-4. doi: 10.1177/0148607114536735 24898210

[16] Desborough JP. The stress response to trauma and surgery. Br J Anaesth. 2000 Jul;85(1): 109-17. doi: 10.1093/bja/85.1.109 10927999

[17] Cusack B, Buggy DJ. Anaesthesia, analgesia, and the surgical stress response. BJA Educ. 2020 Sep;20(9): 321-328. doi: 10.1016/j.bjae.2020.04.006 33456967 PMC7807970

[18] Stoppe C, Goetzenich A, Whitman G, et al. Role of nutrition support in adult cardiac surgery: a consensus statement from an International Multidisciplinary Expert Group on Nutrition in Cardiac Surgery. Crit Care. 2017 Jun 5;21(1):131. doi: 10.1186/s13054-017-1690-5 28583157 PMC5460477

[19] Rowe R, Iqbal J, Murali-Krishnan R, et al. Role of frailty assessment in patients undergoing cardiac interventions. Open Heart. 2014 Feb 1;1(1):e000033. doi: 10.1136/openhrt-2013-000033 25332792 PMC4195918

[20] Kassin MT, Owen RM, Perez SD, et al. Risk factors for 30-day hospital readmission among general surgery patients. J Am Coll Surg. 2012 Sep;215(3): 322-30. doi: 10.1016/j.jamcollsurg.2012.05.024 22726893 PMC3423490

[21] Practice Guidelines for Preoperative Fasting and the Use of Pharmacologic Agents to Reduce the Risk of Pulmonary Aspiration: Application to Healthy Patients Undergoing Elective Procedures: An Updated Report by the American Society of Anesthesiologists Task Force on Preoperative Fasting and the Use of Pharmacologic Agents to Reduce the Risk of Pulmonary Aspiration. Anesthesiology. 2017 Mar;126(3): 376-393. doi: 10.1097/ALN.0000000000001452 28045707

[22] Brady M, Kinn S, Stuart P. Preoperative fasting for adults to prevent perioperative complications. Cochrane Database Syst Rev. 2003;(4):CD004423. doi: 10.1002/14651858.CD00442314584013

[23] Ljungqvist O, Scott M, Fearon KC. Enhanced Recovery After Surgery: A Review. JAMA Surg. 2017 Mar 1;152(3):292-298. doi: 10.1001/jamasurg.2016.4952 28097305

[24] Engelman DT, Ben Ali W, Williams JB, et al. Guidelines for Perioperative Care in Cardiac Surgery: Enhanced Recovery After Surgery Society Recommendations. JAMA Surg. 2019 Aug 1;154(8):755-766. doi: 10.1001/jamasurg.2019.1153 31054241

[25] Low DE, Allum W, De Manzoni G, et al. Guidelines for Perioperative Care in Esophagectomy: Enhanced Recovery After Surgery (ERAS®) Society Recommendations. World J Surg. 2019 Feb;43(2): 299-330. doi: 10.1007/s00268-018-4786-4 30276441

[26] Weimann A, Braga M, Carli F, et al. ESPEN practical guideline: Clinical nutrition in surgery. Clin Nutr. 2021 Jul;40(7): 4745-4761. doi: 10.1016/j.clnu.2021.03.031 34242915

[27] Finnerty CC, Mabvuure NT, Ali A, Kozar RA, Herndon DN. The surgically induced stress response. JPEN J Parenter Enteral Nutr. 2013 Sep;37(5 Suppl):21S-9S. doi: 10.1177/0148607113496117 24009246 PMC3920901

[28] Jakob SM, Stanga Z. Perioperative metabolic changes in patients undergoing cardiac surgery. Nutrition. 2010 Apr;26(4): 349-53. doi: 10.1016/j.nut.2009.07.014 20053534

[29] Kotagal M, Symons RG, Hirsch IB, et al. SCOAP-CERTAIN Collaborative. Perioperative hyperglycemia and risk of adverse events among patients with and without diabetes. Ann Surg. 2015 Jan;261(1): 97-103. doi: 10.1097/SLA.0000000000000688 25133932 PMC4208939

[30] Straatman J, Harmsen AM, Cuesta MA, Berkhof J, Jansma EP, van der Peet DL. Predictive Value of C-Reactive Protein for Major Complications after Major Abdominal Surgery: A Systematic Review and Pooled-Analysis. PLoS One. 2015 Jul 15;10(7):e0132995. doi: 10.1371/journal.pone.0132995 26177542 PMC4503561

[31] Lindenmann J, Fink-Neuboeck N, Koesslbacher M, et al. The influence of elevated levels of C-reactive protein and hypoalbuminemia on survival in patients with advanced inoperable esophageal cancer undergoing palliative treatment. J Surg Oncol. 2014 Nov;110(6): 645-50. doi: 10.1002/jso.23711 24975677

[32] Ferguson M, Capra S, Bauer J, Banks M. Development of a valid and reliable malnutrition screening tool for adult acute hospital patients. Nutrition. 1999 Jun;15(6): 458-64. doi: 10.1016/s0899-9007(99)00084-2 10378201

[33] Di Bella A, Croisier E, Blake C, Pelecanos A, Bauer J, Brown T. Assessing the Concurrent Validity and Interrater Reliability of Patient-Led Screening Using the Malnutrition Screening Tool in the Ambulatory Cancer Care Outpatient Setting. J Acad Nutr Diet. 2020 Jul;120(7): 1210-1215. doi: 10.1016/j.jand.2019.10.015 31892501

[34] Isenring EA, Bauer JD, Banks M, Gaskill D. The Malnutrition Screening Tool is a useful tool for identifying malnutrition risk in residential aged care. J Hum Nutr Diet. 2009 Dec;22(6): 545-50. doi: 10.1111/j.1365-277X.2009.01008.x 20002951

[35] House M, Gwaltney C. Malnutrition screening and diagnosis tools: Implications for practice. Nutr Clin Pract. 2022 Feb;37(1): 12-22. doi: 10.1002/ncp.10801 34897800

[36] Williams DG, Aronson S, Murray S, et al. Validation of the perioperative nutrition screen for prediction of postoperative outcomes. JPEN J Parenter Enteral Nutr. 2022 Aug;46(6): 1307-1315. doi: 10.1002/jpen.2310 34850403

[37] White JV, Guenter P, Jensen G, Malone A, Schofield M; Academy Malnutrition Work Group; A.S.P.E.N. Malnutrition Task Force; A.S.P.E.N. Board of Directors. Consensus statement: Academy of Nutrition and Dietetics and American Society for Parenteral and Enteral Nutrition: characteristics recommended for the identification and documentation of adult malnutrition (undernutrition). JPEN J Parenter Enteral Nutr. 2012 May;36(3): 275-83. doi: 10.1177/0148607112440285 22535923

[38] Mogensen KM, Malone A, Becker P, et al; Malnutrition Committee of the American Society for Parenteral and Enteral Nutrition (ASPEN). Academy of Nutrition and Dietetics/American Society for Parenteral and Enteral Nutrition Consensus Malnutrition Characteristics: Usability and Association With Outcomes. Nutr Clin Pract. 2019 Oct;34(5): 657-665. doi: 10.1002/ncp.10310 31074906

[39] Hummell AC, Cummings M. Role of the nutrition-focused physical examination in identifying malnutrition and its effectiveness. Nutr Clin Pract. 2022 Feb;37(1): 41-49. doi: 10.1002/ncp.10797 34751967

[40] McNicholl T, Dubin JA, Curtis L, et al. Handgrip Strength, but Not 5-Meter Walk, Adds Value to a Clinical Nutrition Assessment. Nutr Clin Pract. 2019 Jun;34(3): 428-435. doi: 10.1002/ncp.10198 30288776

[41] Guyatt GH, Thompson PJ, Berman LB, et al. How should we measure function in patients with chronic heart and lung disease? J Chronic Dis. 1985;38(6):517-24. doi: 10.1016/0021-9681(85)90035-9 4008592

[42] Chainani V, Shaharyar S, Dave K, et al. Objective measures of the frailty syndrome (hand grip strength and gait speed) and cardiovascular mortality: A systematic review. Int J Cardiol. 2016 Jul 15;215:487-93. doi: 10.1016/j.ijcard.2016.04.068 27131770

[43] Fried LP, Tangen CM, Walston J, et al.; Cardiovascular Health Study Collaborative Research Group. Frailty in older adults: evidence for a phenotype. J Gerontol A Biol Sci Med Sci. 2001 Mar;56(3):M146-56. doi: 10.1093/gerona/56.3.m146 11253156

[44] Graham A, Brown CH 4th. Frailty, Aging, and Cardiovascular Surgery. Anesth Analg. 2017 Apr;124(4): 1053-1060. doi: 10.1213/ANE.0000000000001560 27622718 PMC5675521

[45] Prado CM, Ford KL, Gonzaelz MC, et al. Nascent to novel methods to evaluate malnutrition and frailty in the surgical patient. JPEN J Parenter Enteral Nutr. 2023 Feb;47 Suppl 1(Suppl 1): S54-S68. doi: 10.1002/jpen.2420 36468288 PMC9905223

[46] Weerink LBM, van der Hoorn A, van Leeuwen BL, de Bock GH. Low skeletal muscle mass and postoperative morbidity in surgical oncology: a systematic review and meta-analysis. J Cachexia Sarcopenia Muscle. 2020 Jun;11(3): 636-649. doi: 10.1002/jcsm.12529 32125769 PMC7296274

[47] Mertes PM, Kindo M, Amour J, et al. Guidelines on enhanced recovery after cardiac surgery under cardiopulmonary bypass or off-pump. Anaesth Crit Care Pain Med. 2022 Jun;41(3):101059. doi: 10.1016/j.accpm.2022.101059 35504126

[48] Batchelor TJP, Rasburn NJ, Abdelnour-Berchtold E, et al. Guidelines for enhanced recovery after lung surgery: recommendations of the Enhanced Recovery After Surgery (ERAS®) Society and the European Society of Thoracic Surgeons (ESTS). Eur J Cardiothorac Surg. 2019 Jan 1;55(1):91-115. doi: 10.1093/ejcts/ezy301 30304509

[49] Nicholson A, Lowe MC, Parker J, Lewis SR, Alderson P, Smith AF. Systematic review and meta-analysis of enhanced recovery programmes in surgical patients. Br J Surg. 2014 Feb;101(3): 172-88. doi: 10.1002/bjs.9394 24469618

[50] Williams JB, McConnell G, Allender JE, et al. One-year results from the first US-based enhanced recovery after cardiac surgery (ERAS® Cardiac) program. J Thorac Cardiovasc Surg. 2019 May;157(5): 1881-1888. doi: 10.1016/j.jtcvs.2018.10.164 30665758

[51] Smith MD, McCall J, Plank L, Herbison GP, Soop M, Nygren J. Preoperative carbohydrate treatment for enhancing recovery after elective surgery. Cochrane Database Syst Rev. 2014 Aug 14;(8):CD009161. doi: 10.1002/14651858.CD009161.pub2 PMC11060647 25121931

[52] Gianotti L, Biffi R, Sandini M, et al. Preoperative Oral Carbohydrate Load Versus Placebo in Major Elective Abdominal Surgery (PROCY): A Randomized, Placebo-controlled, Multicenter, Phase III Trial. Ann Surg. 2018 Apr;267(4): 623-630. doi: 10.1097/SLA.0000000000002325 28582271

[53] Feguri GR, de Lima PRL, de Cerqueira Borges D, et al. Preoperative carbohydrate load and intraoperatively infused omega-3 polyunsaturated fatty acids positively impact nosocomial morbidity after coronary artery bypass grafting: a double-blind controlled randomized trial. Nutr J. 2017 Apr 20;16(1):24. doi: 10.1186/s12937-017-0245-6 28427403 PMC5397791

[54] Korytkowski MT, Muniyappa R, Antinori-Lent K, et al. Management of Hyperglycemia in Hospitalized Adult Patients in Non-Critical Care Settings: An Endocrine Society Clinical Practice Guideline. J Clin Endocrinol Metab. 2022 Jul 14;107(8):2101-2128. doi: 10.1210/clinem/dgac278 35690958 PMC9653018

[55] Weimann A, Wobith M. ESPEN Guidelines on Clinical nutrition in surgery - Special issues to be revisited. Eur J Surg Oncol. 2022 Oct 13: S0748-7983(22)00694-1. doi: 10.1016/j.ejso.2022.10.002 36280431

[56] Braga M, Gianotti L, Nespoli L, Radaelli G, Di Carlo V. Nutritional approach in malnourished surgical patients: a prospective randomized study. Arch Surg. 2002 Feb;137(2): 174-80. doi: 10.1001/archsurg.137.2.174 11822956

[57] Tepaske R, Velthuis H, Oudemans-van Straaten HM, et al. Effect of preoperative oral immune-enhancing nutritional supplement on patients at high risk of infection after cardiac surgery: a randomised placebo-controlled trial. Lancet. 2001 Sep 1;358(9283):696-701. doi: 10.1016/s0140-6736(01)05836-6 11551575

[58] Senkal M, Zumtobel V, Bauer KH, et al. Outcome and cost-effectiveness of perioperative enteral immunonutrition in patients undergoing elective upper gastrointestinal tract surgery: a prospective randomized study. Arch Surg. 1999 Dec;134(12): 1309-16. doi: 10.1001/archsurg.134.12.1309 10593328

[59] Slim K, Badon F, Vacheron CH, Occean BV, Dziri C, Chambrier C. Umbrella review of the efficacy of perioperative immunonutrition in visceral surgery. Clin Nutr ESPEN. 2022 Apr;48:99-108. doi: 10.1016/j.clnesp.2022.02.015 35331540

[60] Leong JY, van der Merwe J, Pepe S, et al. Perioperative metabolic therapy improves redox status and outcomes in cardiac surgery patients: a randomised trial. Heart Lung Circ. 2010 Oct;19(10): 584-91. doi: 10.1016/j.hlc.2010.06.659 20674497

[61] Heyland DK, Dhaliwal R, Wang M, Day AG. The prevalence of iatrogenic underfeeding in the nutritionally ‘at-risk’ critically ill patient: Results of an international, multicenter, prospective study. Clin Nutr. 2015 Aug;34(4): 659-66. doi: 10.1016/j.clnu.2014.07.008 25086472

[62] Drover JW, Cahill NE, Kutsogiannis J, et al. Nutrition therapy for the critically ill surgical patient: we need to do better! JPEN J Parenter Enteral Nutr. 2010 Nov-Dec;34(6):644-52. doi: 10.1177/0148607110372391 21097764

[63] Stoppe C, Dresen E, Wendt S, et al. Current practices in nutrition therapy in cardiac surgery patients: An international multicenter observational study. JPEN J Parenter Enteral Nutr. 2023 Jul;47(5): 604-613. doi: 10.1002/jpen.2495 36912124

[64] Baker M, Halliday V, Williams RN, Bowrey DJ. A systematic review of the nutritional consequences of esophagectomy. Clin Nutr. 2016 Oct;35(5): 987-94. doi: 10.1016/j.clnu.2015.08.010 26411750 PMC5410167

[65] Berkelmans GHK, Fransen LFC, Dolmans-Zwartjes ACP, et al. Direct Oral Feeding Following Minimally Invasive Esophagectomy (NUTRIENT II trial): An International, Multicenter, Open-label Randomized Controlled Trial. Ann Surg. 2020 Jan;271(1): 41-47. doi: 10.1097/SLA.0000000000003278 31090563

[66] Muscaritoli M, Arends J, Bachmann P, et al. ESPEN practical guideline: Clinical Nutrition in cancer. Clin Nutr. 2021 May;40(5): 2898-2913. doi: 10.1016/j.clnu.2021.02.005 33946039

[67] Arends J, Baracos V, Bertz H, et al. ESPEN expert group recommendations for action against cancer-related malnutrition. Clin Nutr. 2017 Oct;36(5): 1187-1196. doi: 10.1016/j.clnu.2017.06.017 28689670

[68] Stoppe C, Goetzenich A, Whitman G, et al. Role of nutrition support in adult cardiac surgery: a consensus statement from an International Multidisciplinary Expert Group on Nutrition in Cardiac Surgery. Crit Care. 2017 Jun 5;21(1):131. doi: 10.1186/s13054-017-1690-5 28583157 PMC5460477

[69] Berger MM, Berger-Gryllaki M, Wiesel PH, et al. Intestinal absorption in patients after cardiac surgery. Crit Care Med. 2000 Jul;28(7): 2217-23. doi: 10.1097/00003246-200007000-00006 10921543

[70] Marik PE, Zaloga GP. Early enteral nutrition in acutely ill patients: a systematic review. Crit Care Med. 2001 Dec;29(12): 2264-70. doi: 10.1097/00003246-200112000-00005 11801821

[71] Barlow R, Price P, Reid TD, et al. Prospective multicentre randomised controlled trial of early enteral nutrition for patients undergoing major upper gastrointestinal surgical resection. Clin Nutr. 2011 Oct;30(5): 560-6. doi: 10.1016/j.clnu.2011.02.006 21601319

[72] Gabor S, Renner H, Matzi V, et al. Early enteral feeding compared with parenteral nutrition after oesophageal or oesophagogastric resection and reconstruction. Br J Nutr. 2005 Apr;93(4): 509-13. doi: 10.1079/bjn20041383 15946413

[73] Li HN, Chen Y, Dai L, Wang YY, Chen MW, Mei LX. A Meta-analysis of Jejunostomy Versus Nasoenteral Tube for Enteral Nutrition Following Esophagectomy. J Surg Res. 2021 Aug; 264:553-561. doi: 10.1016/j.jss.2021.02.027 33864963

[74] TARGET Investigators, for the ANZICS Clinical Trials Group; Chapman M, Peake SL, Bellomo R, et al. Energy-Dense versus Routine Enteral Nutrition in the Critically Ill. N Engl J Med. 2018 Nov 8;379(19):1823-1834. doi: 10.1056/NEJMoa1811687 30346225

[75] Mazaki T, Ebisawa K. Enteral versus parenteral nutrition after gastrointestinal surgery: a systematic review and meta-analysis of randomized controlled trials in the English literature. J Gastrointest Surg. 2008 Apr;12(4): 739-55. doi: 10.1007/s11605-007-03621 17939012

[76] Heidegger CP, Berger MM, Graf S, et al. Optimisation of energy provision with supplemental parenteral nutrition in critically ill patients: a randomised controlled clinical trial. Lancet. 2013 Feb 2;381(9864):385-93. doi: 10.1016/S0140-6736(12)61351-8 23218813

[77] Wischmeyer PE, Hasselmann M, Kummerlen C, et al. A randomized trial of supplemental parenteral nutrition in underweight and overweight critically ill patients: the TOP-UP pilot trial. Crit Care. 2017 Jun 9;21(1):142. doi: 10.1186/s13054-017-1736-8 28599676 PMC5466764

[78] Manzanares W, Langlois PL, Dhaliwal R, Lemieux M, Heyland DK. Intravenous fish oil lipid emulsions in critically ill patients: an updated systematic review and meta-analysis. Crit Care. 2015 Apr 16;19(1):167. doi: 10.1186/s13054-015-0888-7 25879776 PMC4404291

[79] Ferrie S, Herkes R, Forrest P. Nutrition support during extracorporeal membrane oxygenation (ECMO) in adults: a retrospective audit of 86 patients. Intensive Care Med. 2013 Nov;39(11): 1989-94. doi: 10.1007/s00134-013-3053-2 23949702

[80] Umezawa Makikado LD, Flordelís Lasierra JL, Pérez-Vela JL, et al. Early enteral nutrition in adults receiving venoarterial extracorporeal membrane oxygenation: an observational case series. JPEN J Parenter Enteral Nutr. 2013 Mar;37(2): 281-4. doi: 10.1177/0148607112451464 22750804

[81] Karpasiti T. A Narrative Review of Nutrition Therapy in Patients Receiving Extracorporeal Membrane Oxygenation. ASAIO J. 2022 Jun 1;68(6):763-771. doi: 10.1097/MAT.0000000000001540 34324446

